# Early Immune Signature Features, Including TLR2 and TLR4 Expression, Are Associated with Complete Remission After CD19 CAR-T Cell Therapy

**DOI:** 10.3390/ph19050671

**Published:** 2026-04-25

**Authors:** Serena Di Iasio, Chiara Di Nunzio, Elisabetta De Santis, Concetta Stella, Daniela Valente, Dalila Salvatore, Emanuela Merla, Grazia Dell’Olio, Costanzo Padovano, Mattia Colucci, Gaja Bruno, Barbara Pasculli, Mario Caldarelli, Paola Parrella, Giovanni Gambassi, Rossella Cianci, Angelo M. Carella, Vincenzo Giambra

**Affiliations:** 1 Hematopathology Unit, Institute for Stem Cell Biology, Regenerative Medicine and Innovative Therapeutics (ISBReMIT), Fondazione IRCCS “Casa Sollievo della Sofferenza”, Viale Padre Pio, 7, 71013 San Giovanni Rotondo, Italy; s.diiasio@operapadrepio.it (S.D.I.); c.dinunzio@operapadrepio.it (C.D.N.); e.desantis@operapadrepio.it (E.D.S.); c.stella@operapadrepio.it (C.S.); costanzo.padovano@operapadrepio.it (C.P.); m.colucci@operapadrepio.it (M.C.); g.bruno@operapadrepio.it (G.B.); v.giambra@operapadrepio.it (V.G.); 2 Department of Hematology and Stem Cell Transplant Unit, Fondazione IRCCS “Casa Sollievo della Sofferenza”, Viale Capuccini, 1, 71013 San Giovanni Rotondo, Italy; d.valente@operapadrepio.it (D.V.); d.salvatore@operapadrepio.it (D.S.); e.merla@operapadrepio.it (E.M.); g.dellolio@operapadrepio.it (G.D.); 3 Laboratory of Oncology, Fondazione IRCCS “Casa Sollievo della Sofferenza”, Viale Cappuccini, 1, 71013 San Giovanni Rotondo, Italy; b.pasculli@operapadrepio.it (B.P.); pparrella@operapadrepio.it (P.P.); 4 Department of Translational Medicine and Surgery, Catholic University of Sacred Heart, Largo Agostino Gemelli, 8, 00168 Rome, Italy; mario.caldarelli01@icatt.it (M.C.); giovanni.gambassi@unicatt.it (G.G.); 5 Fondazione Policlinico Universitario A. Gemelli, Istituto di Ricerca e Cura a Carattere Scientifico (IRCCS), Largo Agostino Gemelli, 8, 00168 Rome, Italy

**Keywords:** DLBCL, CAR-T cells, Toll-like receptors 2 and 4 (TLR2; TLR4), complete remission (CR), flow cytometry, biomarkers

## Abstract

**Background/Objectives:** CD19-directed chimeric antigen receptor T (CAR-T) cell therapy induces profound immune remodeling. Nonetheless, biomarkers predicting complete remission (CR) remain poorly defined. We characterized longitudinal cytokine and immune-cell dynamics after CAR-T infusion and identified early immunological features associated with CR. **Methods:** Longitudinal immune profiling was performed in 18 patients with non-Hodgkin lymphoma, including 14 with relapsed/refractory diffuse large B-cell lymphoma treated with anti-CD19 CAR-T cells. Peripheral blood was collected at the baseline and days 7, 14, 21, 28, and 60 post-infusion. Multiparameter flow cytometry quantified lymphoid and myeloid subsets and Toll-like receptor (TLR)2 and TLR4 expression. Serum cytokines were measured by multiplex assays. Machine-learning-based feature selection identified variables associated with CR. **Results:** Two inflammatory waves were observed. The first, at day 7, featured elevated IL-6, IL-10, IFN-α, IFN-γ, and TNF-α, accompanied by increased CD4^+^ T cells, HLA-DR^high^ classical monocytes, and non-classical monocytes. The second, at days 21–28, showed increased IL-5, IL-6, IL-12, IFN-γ, and GM-CSF, with expansion of CD4^+^ and CD8^+^ T cells, regulatory T cells, NK-T cells, and non-classical monocytes. TLR2 expression was significantly upregulated at day 7 on T-cell subsets and on classical and intermediate monocytes. An exploratory feature-selection analysis identified baseline and day-7 TLR2 and TLR4 expression on lymphoid and myeloid cells, early IFN-γ levels, and monocyte frequencies as variables associated with CR. **Conclusions:** Together, these data show that anti-CD19 CAR-T therapy induces two coordinated waves of cytokine release and immune-cell activation. Moreover, the findings suggest that early modulation of innate immune features, particularly TLR2 expression, is associated with complete remission, although these biomarker relationships remain exploratory and require validation in larger cohorts.

## 1. Introduction

Chimeric antigen receptor (CAR) T-cell therapy has become an established treatment for several hematological malignancies, particularly relapsed or refractory B-cell lymphomas, achieving high rates of durable complete remission (CR) [1,2]. Following infusion, CAR-T cells induce a profound remodeling of the immune system characterized by intense cytokine release and activation of both innate and adaptive immune compartments [3]. This inflammatory surge underlies cytokine release syndrome (CRS) and immune effector cell-associated neurotoxicity syndrome (ICANS), the two most frequent and potentially severe toxicities, which are largely driven by myeloid cell activation and excessive production of pro-inflammatory mediators [4]. Although CRS and ICANS represent the most evident clinical manifestations of this immune activation, the same inflammatory and innate sensing pathways may also critically influence antitumor efficacy and the achievement of CR [5].

To date, numerous soluble factors and cellular subsets have been proposed as biomarkers of CAR-T-induced inflammation and clinical outcome, yet early predictors of complete remission remain incompletely defined [3,6,7,8,9]. Moreover, machine-learning approaches have recently been proposed as a promising framework to address this challenge, offering the potential to integrate high-dimensional immune datasets and identify candidate biomarkers in an unbiased manner [10]. Interestingly, Toll-like receptors (TLRs) have also been widely investigated as regulators of antitumor immunity and as potential therapeutic targets in cancer immunotherapy [11,12]. TLRs are key components of innate immune sensing, acting as pattern-recognition receptors for pathogen-associated and damage-associated molecular patterns and orchestrating cytokine production through signaling mediated by the Toll/IL-1 receptor (TIR) domain [13]. TLR2 and TLR4 are especially important when considering CAR-T cell therapy, as they detect pathogen-associated and damage-associated molecular patterns (PAMPs and DAMPs) that are released when tumor cells are killed and tissue is damaged. The signals produced by these receptors activate the MyD88-dependent pathway leading to the activation of NF-κB and interferon regulatory factors [13]. The resulting inflammatory cytokines, such as IL-6, TNF-α, IL-12, and type I interferon, are critically involved in CAR-T cell expansion, differentiation, and persistence, as well as playing a role in the development of CRS [3,5].

Recent evidence has also shown that the activation of myeloid cells is not simply a result of the cytotoxicity mediated by CAR-T cell therapy, but rather a factor impacting both the level of toxicity and the antitumor efficacy of CAR-T cells [14]. Cytokines released from monocytes, such as IL-12 and type I interferons, may enhance T-cell effector functions and create a Th1-skewed environment, which may promote both CAR-T cell expansion and cytotoxicity. Dysregulated (or aberrant) signaling from TLRs could also lead to immune exhaustion or immunosuppressive feedback loops [11,12,15]. Additionally, TLR2 and TLR4 are present not only in monocytes and macrophages, but also on activated T-cell subsets, where they can affect costimulatory signaling, metabolic fitness, and/or cytokine production [16]. Therefore, variations in TLR2/TLR4 expression may reflect differences in the baseline innate immune system activity [17].

From this framework, we hypothesize that TLR2 and TLR4 expression (both baseline and early post-infusion) could be used as integrative biomarkers for host immune response and for early innate-adaptive cross-talk, and be associated with the likelihood of achieving complete remission. Specifically, we propose that enhanced early TLR-driven signaling may be associated with a coordinated interferon-rich cytokine program and effective CAR-T cell expansion, both of which will favor durable tumor clearance. In order to test our hypothesis, we have conducted a longitudinal immune profiling of cytokines, immune cell subsets, and TLR2/TLR4 expression in patients receiving CD19 CAR-T therapy, and assessed their relationship with complete remission at day 100 from infusion.

## 2. Results

To identify potential biomarkers associated with the early inflammatory response to CD19 CAR-T therapy, we conducted longitudinal immune profiling using multiparameter flow cytometry in 18 patients with non-Hodgkin lymphoma (NHL), including 14 with relapsed/refractory Diffuse Large B-Cell Lymphoma (DLBCL R/R), one with relapsed/refractory high-grade non-Hodgkin lymphoma (NHL-HG R/R), one with high-grade non-Hodgkin lymphoma (NHL-HG) and two with non-Hodgkin lymphoma (NHL), treated with axicabtagene ciloleucel [18] CD19 CAR-T therapy (Table S1). Peripheral blood was collected at the baseline (day 0) and at days 7, 14, 21, 28, and 60 post-infusion of CAR-T cells, and at days 0 and 28 for next-generation sequencing genetic profiling using the Ion Torrent Oncomine panel (Table S2).

**Figure 1:**
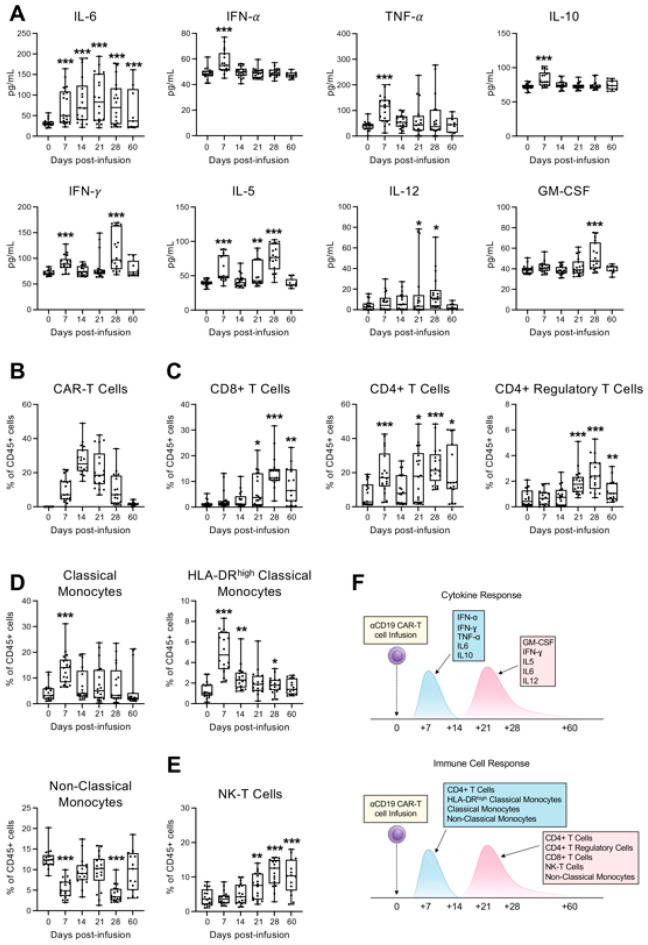
Longitudinal immune profiling of 18 patients with non-Hodgkin lymphoma (NHL), treated with axicabtagene ciloleucel CD19 CAR-T therapy. (**A**) Plasma level of IL-5, IL-6, IL-10, IL-12, IFN-α, IFN-γ, TNF-α and GM-CSF in patients following CD19 CAR-T cell infusion. The concentrations of indicated human soluble cytokines and chemokines in the plasma of cancer patients (*N* = 18) were measured at baseline (day 0) and on days 7, 14, 21, 28, and 60 following CD19 CAR-T cell infusion. Quantification was performed simultaneously, using flow cytometry with MACSPlex Capture Beads (Miltenyi Biotec Inc., Bergisch Gladbach, Germany). Data are shown as median with interquartile range. Statistical significance compared to baseline is indicated (* *p* < 0.05, ** *p* < 0.01, *** *p* < 0.001; Student’s *t*-test between each post-infusion time point and baseline (day 0)). (**B**–**E**) Multiparameter flow cytometry analysis of indicated immune cell populations in peripheral blood from 14 patients at baseline (day 0) and on days 7, 14, 21, and 28 after CD19 CAR-T cell infusion. Peripheral blood mononuclear cells (PBMCs) from each individual sample were labeled after red cell lysis, using the reported panel of fluorophore-conjugated antibodies against lineage-specific cell surface markers in order to identify the CAR-T cells (**B**); CAR-negative T cell subsets (**C**); HLA-DR^high^ Classical and Non-Classical Monocytes (**D**); and Natural killer (NK) T-cells (**E**). Data are shown as median with interquartile range. Statistical significance compared to baseline is indicated (* *p* < 0.05, ** *p* < 0.01, *** *p* < 0.001; Student’s *t*-test between each post-infusion time point and baseline (day 0)). (**F**) Schematic timeline illustrating the blood sampling schedule and longitudinal monitoring following CD19 CAR-T cell infusion, highlighting the integration of cytokine and immune cell profiling.

We first quantified the plasma levels of soluble cytokines and chemokines to capture temporal patterns in the innate immune response. A distinct biphasic cytokine profile emerged: an early peak at day 7 characterized by elevated IL-6, IL-10, IFN-α, IFN-γ, and TNF-α, followed by a secondary wave between days 21–28 marked by increased IL-5, IL-6, IL-12, IFN-γ, and GM-CSF (Figure 1A and Figure S1), which was consistent with a multi-phase inflammatory process [4]. To explore cellular immunophenotypic changes, we designed three multiparameter flow cytometry panels (Tables S3–S6) for tracking CAR-T cells and the major immune cell subsets, including T, B, NK, monocytes and macrophages.

As previously reported [19], CAR-T cells expanded in vivo post-infusion, with peak levels around day 14 (Figure 1B). Concomitantly, we observed an early rise in CAR-negative CD4^+^ T cells and HLA-DR^high^ classical and non-classical monocytes at day 7, followed by broader immune activation between days 21–28. This later phase included increased frequencies of CAR-negative CD4^+^ and CD8^+^ T cells, CD4^+^ regulatory T cells, NK-T cells, and non-classical monocytes (Figure 1C–E, Figures S2 and S3), suggesting a coordinated innate and adaptive response in two phases (Figure 1F) that may be critical in shaping treatment outcomes and toxicities.

**Figure 2:**
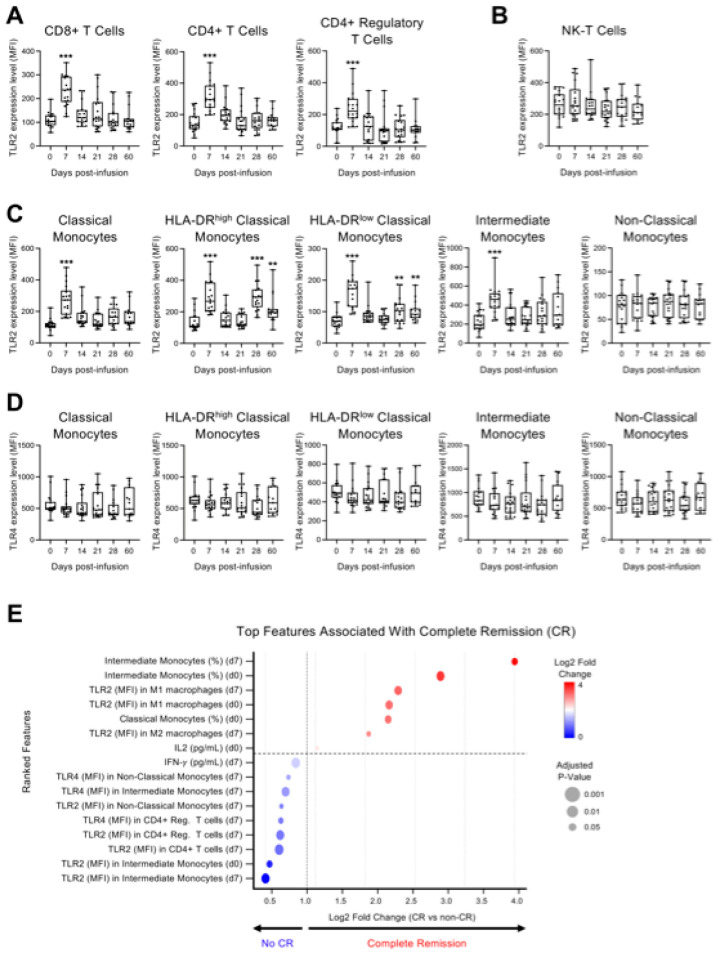
TLR2 and TLR4 expression in immune cell subsets and their association with complete remission (CR) following CD19 CAR-T cell therapy. (**A**–**D**) Flow cytometry assessment of TLR2 and TLR4 expression in the indicated cell lineages in peripheral blood mononuclear cells (PBMCs) from patients at baseline (day 0) and on days 7, 14, 21, and 28 after CD19 CAR-T cell infusion. TLR2 and TLR4 expression was measured by mean fluorescence intensity (MFI) in CD4^+^ T cells, CD8^+^ T cells and CD4^+^ regulatory T cell subsets (**A**); Natural killer (NK-T) T cells (**B**); and monocyte subsets (**C**,**D**). Data are shown as median with interquartile range. Statistical significance compared to baseline is indicated (** *p* < 0.01, *** *p* < 0.001; Student’s *t*-test between each post-infusion time point and baseline (day 0)). (**E**) Top features associated with complete remission (CR) at day 100 after CD19 CAR-T cell therapy. A ranked feature-importance plot displays the top 16 biomarkers identified using logistic regression with L2 regularization (glmnet), supported by random forest-based feature ranking. Only variables that were consistently ranked among the top features by both approaches were retained. Features include baseline (day 0) cytokine concentrations (e.g., IL-2), immune cell frequencies, and expression levels of TLR2 and TLR4 measured as mean fluorescence intensity (MFI) across multiple immune subsets, including monocytes, macrophages, and T-cell populations, assessed at baseline and day 7 post-infusion. Features are ordered by log2 fold change between CR and non-CR groups. Circle size represents adjusted *p*-values (Benjamini–Hochberg FDR), and color indicates direction of association (red, higher in CR; blue, higher in non-CR). Exact log2 fold-change values and corresponding adjusted *p*-values for all evaluated features are reported in Supplementary Table S7.

TLR2 and TLR4 play a critical role in the progression of inflammation and the development of a cytokine storm [20]. Therefore, given the elevation of inflammatory cytokines observed post-infusion, we further analyzed the expression of TLR2 and TLR4 in various immune subsets by flow cytometry (Figure 2). Interestingly, TLR2 expression, measured by mean fluorescence intensity (MFI), was significantly upregulated in CAR-negative CD4^+^ and CD8^+^ T cells, as well as in CD4^+^ regulatory T cells, with peak levels observed on day 7 (Figure 2A). In contrast, no significant changes were detected in NK-T cells (Figure 2B). Similarly, classical and intermediate monocytes exhibited a peak of TLR2 expression at day 7, while both HLA-DR^high^ and HLA-DR^low^ classical monocytes showed significant upregulation not only at day 7 but also between days 28 and 60 (Figure 2C), suggesting prolonged engagement of monocyte subsets in the post-infusion inflammatory response. In contrast, no significant changes were observed in TLR4 expression, particularly within classical and intermediate monocyte populations (Figure 2D).

We next performed a feature-ranking analysis integrating genetic mutations and variants identified by targeted genetic profiling using the Ion Torrent Oncomine panel (Table S2), together with cytokine levels, immune-cell frequencies, and TLR expression, to identify the variables associated with the achievement of complete remission (CR) at day 100 after CD19 CAR-T cell therapy (Figure 2E). Targeted gene profiling was included to evaluate whether the detected tumor-intrinsic genomic alterations contributed to the treatment response. However, no recurrent mutations or variants showed an association with CR and therefore did not rank among the top highlighted features. In contrast, the exploratory candidate features associated with CR included the frequency of intermediate monocytes at the baseline and at day 7, as well as TLR2 expression in M1 macrophages at both time points. Elevated TLR2 expression in CD4^+^ T cells and CD4^+^ regulatory T cells at day 7, together with TLR4 expression in intermediate and non-classical monocytes, as well as early IFN-γ levels at day 7 and baseline classical monocyte frequencies, contributed to the top-ranked set of variables that differed between groups in the reported exploratory analysis.

## 3. Discussion

In this study, we performed a longitudinal and integrated analysis of cytokine dynamics, immune-cell remodeling, and Toll-like receptor (TLR) expression following CD19 CAR-T cell therapy, identifying early innate and adaptive immune features associated with the achievement of complete remission (CR). Our data demonstrate a temporally structured inflammatory response, with an early interferon-associated phase occurring around day 7, followed by later adaptive immune expansion between days 21 and 28. This temporal sequence is consistent with the known kinetics of CAR-T cell expansion and immune reconstitution [19] and supports the concept that early immune activation patterns may influence downstream immune recovery and clinical outcomes.

A central observation of our study is the early upregulation of TLR2 across lymphoid and myeloid compartments, including CD4^+^ and CD8^+^ T cells, regulatory T cells, and classical and intermediate monocytes, with comparatively modest modulation of TLR4. TLR2 and TLR4 are key pattern-recognition receptors that integrate microbial-derived and damage-associated signals and amplify NF-κB and interferon signaling pathways [21,22]. The peak of TLR2 expression at day 7 coincided with maximal levels of IFN-α, IFN-γ, TNF-α, and IL-6, suggesting that TLR-associated signaling may occur alongside early inflammatory activation following CAR-T infusion. However, given the observational design of this study, these findings should not be interpreted as evidence of a direct mechanistic role of TLR-associated signaling in mediating antitumor responses. An alternative explanation is that increased TLR expression reflects a broader activation state of innate and adaptive immune cells during early immune reconstitution, rather than a specific driver of therapeutic efficacy.

Although we incorporated targeted next-generation sequencing (NGS) to evaluate whether the detected tumor-intrinsic genetic alterations might contribute to treatment response, no recurrent mutations or variants were significantly associated with CR, and genomic features did not rank among the top features in the machine-learning-based analysis. In contrast, baseline and early post-infusion immunological parameters, particularly those related to the myeloid compartment and TLR signaling, emerged as variables that were associated with the clinical outcome in exploratory analyses. Frequencies of intermediate monocytes, TLR2 expression in M1 macrophages and T-cell subsets, TLR4 expression in intermediate and non-classical monocytes, and early IFN-γ levels ranked among the top features associated with patients who achieved CR from those who did not (CR vs. non-CR). These findings are broadly consistent with prior work in r/r B-NHL patients receiving CAR-T therapy, where logistic regression-based prediction models have similarly highlighted pre-infusion T-cell and monocyte parameters as candidate determinants of clinical response [23]. Nonetheless, the observed associations should be interpreted cautiously as correlational findings that may reflect underlying immune competence, systemic inflammation, or early CAR-T expansion dynamics, rather than independent predictors of treatment response. In our cohort, no significant associations were observed between TLR2- and TLR4-related immune signatures and the severity of cytokine release syndrome (CRS) or immune effector cell-associated neurotoxicity syndrome (ICANS), most likely due to the limited number of patients experiencing high-grade CRS or ICANS. Nonetheless, these findings indicate that the pre-existing innate immune tone and its early amplification after CAR-T infusion may represent the correlates of effective antitumor responses, although confirmation in larger, covariate-adjusted cohorts will be required.

Recent clinical and translational studies have highlighted the gut microbiome as a major upstream regulator of CAR-T cell efficacy. Stein-Thoeringer et al. demonstrated that preservation of a non-antibiotic-disrupted microbiome is associated with superior expansion, persistence, and clinical responses to CD19 CAR-T cells [24], while Prasad et al. showed that the loss of microbial metabolic output following broad-spectrum antibiotic exposure correlates with inferior outcomes [25]. These findings provide a biologically plausible framework within which our observations may be interpreted, as microbial-associated molecular patterns and microbiota-derived metabolites are key ligands and modulators of TLR2 and TLR4 signaling. Inter-individual variation in microbiome composition and function may therefore shape the baseline and early post-infusion TLR expression in myeloid and lymphoid cells, modulate interferon and cytokine programs, and ultimately influence the probability of achieving CR. However, because microbiome composition was not directly measured in our cohort, any linkage between microbiome variation and TLR-related immune features remains speculative and should be formally tested in future studies incorporating integrated microbiome analyses.

Finally, some limitations of this study should be acknowledged. First, the cohort size was relatively small and derived from a single center, limiting the statistical power and the generalizability of the identified biomarker signatures. Although machine-learning-based feature selection was applied, independent validation in larger, multi-center cohorts and across different CAR-T products is required for any predictive conclusions. Second, multiple immune parameters were evaluated across several time points, using descriptive longitudinal comparisons without formal correction for multiple testing in all analyses; therefore, the possibility of false-positive findings cannot be excluded, and these observations should be interpreted cautiously. Third, immune profiling was restricted to peripheral blood and may not fully capture immune dynamics within lymphoid tissues or the tumor microenvironment, where critical interactions between CAR-T cells, myeloid cells, and malignant B cells occur. Fourth, the observational nature of the study precludes causal inference; functional studies will be necessary to determine whether the modulation of TLR2 or TLR4 signaling directly influences CAR-T cell expansion, persistence, and cytotoxic function. Fifth, given the limited sample size and the exploratory nature of this study, analyses were not powered to support comprehensive multivariable modeling incorporating established clinical determinants of CAR-T response, such as the baseline tumor burden, CAR-T cell expansion kinetics, or rates of high-grade CRS/ICANS, and therefore the identified molecular signatures should be considered hypothesis-generating only, which is not suitable at this stage for clinical decision-making or patient stratification. Finally, while targeted NGS did not reveal outcome-associated tumor genetic features in this cohort, broader genomic profiling, along with direct assessment of microbiome composition, metabolic output, and host genetic variation in innate immune pathways, may further refine predictive models and should be incorporated into future integrative analyses.

In conclusion, our data demonstrate that CD19 CAR-T cell therapy was associated with coordinated temporal changes in cytokine production and immune-cell activation, and early innate immune signatures—including TLR2 expression across myeloid and T-cell subsets—were among the exploratory features associated with complete remission. Together with emerging evidence on the role of the gut microbiome in CAR-T outcomes [24,25], these findings are consistent with a speculative model in which host–microbe interactions, innate immune activation, and adaptive immune reconstitution may collectively shape therapeutic outcomes. However, the present data support association rather than causation, and further mechanistic and validation studies will be required to clarify the functional relevance of these immune features.

## 4. Materials and Methods

### 4.1. Human Samples and Blood Cell Counts

The study was conducted following approval by the Research Ethics Committee of the Fondazione I.R.C.C.S. Casa Sollievo della Sofferenza (approval code 50/CE/2024, issued on 17 April 2024). Peripheral blood specimens were obtained from 18 patients diagnosed with non-Hodgkin lymphoma (NHL), including 14 individuals with relapsed or refractory Diffuse Large B-Cell Lymphoma (DLBCL R/R) who were treated with axicabtagene ciloleucel. Written informed consent was secured from all participants in accordance with the Declaration of Helsinki. All participants were Caucasian and from the same geographic area, and the median age was 52 years. Moreover, the study cohort consisted of 18 patients, including 12 males (67%) and 6 females (33%). Cytokine release syndrome (CRS) occurred in 11 patients (61%), while immune effector cell-associated neurotoxicity syndrome (ICANS) was observed in 3 patients (17%). At day 100 post-infusion, 8 patients (44%) achieved complete remission (CR), 6 (33%) achieved partial remission (PR), 3 (17%) had progressive disease (PD), and 1 patient was not evaluable. For outcome-based analyses, patients were grouped as CR (*n* = 8) versus non-CR (PR + PD; *n* = 9), excluding the non-evaluable case. Peripheral blood samples were collected longitudinally at the baseline (day 0, prior to infusion) and at days 7, 14, 21, 28, and 60 after CAR-T cell administration. Detailed demographic and clinical characteristics are provided in Supplementary Table S1. Complete blood counts and routine hematological parameters were measured at the Fondazione I.R.C.C.S. Casa Sollievo della Sofferenza research hospital, using the Sysmex XT-4000i automated hematology analyzer (Sysmex Corporation, Kobe, Japan) according to standard laboratory protocols. The corresponding data are presented in Supplementary Figure S2.

### 4.2. Isolation of Peripheral Blood Mononuclear Cells (PBMCs)

Peripheral blood mononuclear cells (PBMCs) were separated from whole blood by density-gradient centrifugation using Ficoll-Paque PLUS (GE Healthcare, Chicago, IL, USA). Briefly, 6 mL of peripheral blood collected in EDTA tubes was diluted 1:1 with phosphate-buffered saline (PBS, pH 7.4) supplemented with 0.05 M EDTA (Invitrogen, Carlsbad, CA, USA). The diluted blood (12 mL) was carefully layered onto 24 mL of Ficoll-Paque PLUS and centrifuged at 400× *g* for 30 min at room temperature using a swinging-bucket rotor (Eppendorf, Hamburg, Germany), with both the acceleration and brake settings turned off. Following centrifugation, the mononuclear cell layer located at the plasma–Ficoll interface was gently aspirated and transferred to a clean tube. Cells were washed with PBS containing EDTA and centrifuged at 250× *g* for 10 min to remove residual Ficoll and platelets. To eliminate the remaining erythrocytes, the cell pellet was resuspended in ammonium chloride-based red blood cell lysis buffer (Stemcell Technologies, Vancouver, BC, Canada) and incubated for 10 min at room temperature under gentle agitation. After lysis, PBMCs were washed again with PBS-EDTA, pelleted, and resuspended in freezing medium consisting of fetal calf serum (FCS; Invitrogen) supplemented with 10% dimethyl sulfoxide (DMSO; Thermo Fisher Scientific, Waltham, MA, USA). Cells were gradually frozen and stored in liquid nitrogen until further experimental use.

### 4.3. Assessment of Cytokine and Chemokine Levels

The plasma samples of patients collected at the baseline (day 0) and at days 7, 14, 21, 28, and 60 post-infusion of CAR-T cells were isolated from whole blood using Ficoll-Paque as previously described. The MACSPlex Cytokine 12 Kit (cat. 130-099-169, Miltenyi Biotec., Bergisch Gladbach, Germany) was employed for determining the concentrations of the following cytokines: GM-CSF, IFN-α, IFN-γ, IL-2, IL-4, IL-5, IL-6, IL-9, IL-10, IL-12, IL-17 and TNF-α. A total of 1 mL of each plasma sample was incubated overnight in the dark on a shaking platform (1400 rpm) at room temperature with 20 µL of MACSPlex Capture Beads and treated according to the manufacturer’s recommendations. After staining, the beads coated with capture antibodies against the reported soluble analytes were detected using FACS Canto2 (Becton Dickinson, Franklin Lakes, NJ, USA). The flow cytometry data were assessed using FlowJo 10.10.1 (Becton Dickinson, Franklin Lakes, USA) and GraphPad-Prism 10.4.2 software for the visualization and statistical analysis of the quantitative data.

### 4.4. Flow Cytometry Assays

To perform the indicated multiparameter flow cytometry assay, PBMCs were isolated as previously described and stained with the multi-color antibody panels reported in Supplementary Tables S3–S5 in DPBS with BD Horizon Brilliant Stain Buffer (Becton Dickinson) for 20 min at room temperature. The LIVE/DEAD™ Fixable Near-IR dead cell stain kit (cat. L34975, ThermoFisher Scientific, Waltham, MA, USA) was included to identify viable cells. All flow cytometry analyses were performed on FACS Canto2 (Becton Dickinson) or MoFlo Astrios cell sorter (Beckman Coulter Inc., Brea, CA, USA). The instrument performance was routinely verified using standard calibration procedures, and compensation was established using single-stained controls. Fluorescence minus one (FMO) controls were applied to define the gating boundaries for all markers. Doublets were excluded based on side scatter height (SSC-H) versus side scatter area (SSC-A), and dead cells were removed using the viability dye.

Leukocytes were first identified as CD45^+^ events. Within this population, CAR-T cells were defined as CD19^−^CD3^+^ cells expressing the CAR construct (FMC63^+^). The remaining CD19^−^CD3^+^FMC63^−^ T-cell compartment was subdivided into CD8^+^ T cells (CD4^−^CD8^+^), CD4^+^ T cells (CD4^+^CD8^−^), and regulatory T cells (Tregs), defined as CD4^+^CD8^−^CD127^−^ cells. Natural killer T (NKT) cells were identified as CD3^+^CD56^+^ cells, whereas natural killer (NK) cells were defined as CD3^−^CD56^+^CD19^−^ lymphocytes. B lymphocytes were gated as CD3^−^CD19^+^HLA-DR^+^ cells, and plasma cells were distinguished within the B-cell compartment by high CD38 expression (CD38^high^).

Monocyte subsets were characterized based on CD14 and CD16 expression as classical (CD14^+^CD16^−^), intermediate (CD14^+^CD16^+^), and non-classical (CD14^dim^CD16^+^) monocytes. Classical monocytes were further stratified into HLA-DR^high^ and HLA-DR^low^ fractions. Within the CD14^+^CD16^+^CD163^−^ myeloid compartment, macrophage polarization states were evaluated by identifying CD80^+^ cells as M1-like macrophages and CD80^−^ cells as M2-like macrophages. Gating was applied consistently across samples using standardized templates, and all analyses were performed blindly to clinical outcomes.

### 4.5. Next-Generation Sequencing (NGS) Genetic Profiling

Peripheral blood samples (2 mL) from cancer patients were collected at the time of CAR-T cell infusion and 28 days post-infusion into EDTA-containing tubes. DNA isolation was performed using the QIAamp DNA Mini Kit (Cat. QG51304, Qiagen, Venlo, The Netherlands). DNA quantification was subsequently carried out using the Qubit™ dsDNA Quantification Assay Kits (Cat. Q32851, ThermoFisher Scientific, Waltham, USA) with Qubit™ Assay Tubes (Cat. Q32856, ThermoFisher Scientific, Waltham, USA) on a Qubit™ 4 Fluorometer (Cat. Q33226, ThermoFisher Scientific, Waltham, USA). Quantified DNA samples were processed using the Oncomine™ Childhood Cancer Research Assay (OCCRA) (Cat. A36486, ThermoFisher Scientific, Waltham, USA) for library preparation and sequencing. The process involves constructing an Ion Torrent adapter-ligated library with the Ion AmpliSeq™ Library Kit Plus (Cat. 4488990, ThermoFisher Scientific, Waltham, USA) through end-repair and ligation of P1, Ion Xpress™ Barcode Adapters (Cat. 4471250, ThermoFisher Scientific, Waltham, USA), following manufacturer’s guidelines (Cod. MAN0017117, ThermoFisher Scientific, Waltham, USA). Library purification was performed with AMPure XP (Cat. A63881, Beckman Coulter Inc., Brea, USA) and a DynaMag™-2 Magnet (Cat. 12321D, ThermoFisher Scientific, Waltham, USA). Following preparation, libraries were quantified and their size distribution was determined using the 2200 TapeStation System (Cat. G2964AA, Agilent Technologies, Inc., Santa Clara, CA, USA). DNA quantification for pooling was performed using the Qubit™ dsDNA HS Assay Kit (Cat. Q32851, ThermoFisher Scientific, Waltham, USA). Libraries were pooled following manufacturer’s guidelines and sequenced on the Ion GeneStudio™ S5 System (Cat. A38194, ThermoFisher Scientific, Waltham, USA) after initialization using Ion S5™ Sequencing Solutions (Part No. A27767, ThermoFisher Scientific, Waltham, USA) and Ion S5™ Sequencing Reagents (Part No. A27768, ThermoFisher Scientific, Waltham, USA) kits. Sequencing data analysis, including variant calling and annotation, was managed using Torrent Suite and Ion Reporter™ v5.20 software. Filtering and validation steps were applied. Oncomine Extended (5.20) filter chains were utilized for data processing.

### 4.6. Statistical Analysis

For the longitudinal descriptive analyses shown in Figure 1 and Figure 2, statistical comparisons were performed between each post-infusion time point and baseline (day 0), using pairwise Student’s *t*-tests. Given the exploratory design, the limited cohort size, and the biologically guided comparisons versus the baseline, formal correction for multiple comparisons was not applied. Accordingly, these results should be interpreted as descriptive and hypothesis-generating, requiring validation in larger independent cohorts. Statistical analyses were performed using R (version 4.5) and GraphPad Prism (version 10.4.2), with the choice of test determined by data distribution and variance. A *p*-value < 0.05 was considered to be statistically significant.

### 4.7. Feature-Ranking Analysis

All statistical and machine-learning analyses were performed in R (version 4.3.1; R Foundation for Statistical Computing, Vienna, Austria). The analysis was conducted on a dataset comprising cytokine concentrations, immune-cell frequencies, TLR2/TLR4 MFI values, and targeted genomic features measured at the baseline and day 7 post-infusion across 18 patients (11 achieving complete remission [CR] at day 100, 7 non-CR). Given the limited sample size relative to the number of input features, this analysis was framed and is reported throughout as explicitly exploratory; no predictive model was trained or tested, and the results are interpreted solely as hypothesis-generating feature rankings.

Prior to analysis, features with more than 20% missing values across samples were excluded. For the remaining missing values, median imputation was applied separately within each clinical outcome group (CR vs. non-CR) to avoid data leakage, implemented using the mice package (version 3.16.0) [26]. Multicollinearity among input features was assessed by computing pairwise Spearman correlation coefficients. Feature selection was primarily performed using logistic regression with L2 (ridge) regularization, implemented through the glmnet package (version 4.1-8) [27]. This analytical combination has been previously applied in analogous clinical settings involving CAR-T cell therapy [28]. Regularization was applied to reduce overfitting and improve model stability, given the limited sample size. All continuous features were standardized to zero mean and unit variance prior to fitting. The regularization hyperparameter lambda was selected via leave-one-out cross-validation (LOOCV), chosen over k-fold approaches given the small cohort size (*n* = 18), to maximize the use of the available training data at each iteration. Feature-level *p*-values were derived by permutation testing (1000 permutations) and adjusted for multiple testing using the Benjamini–Hochberg FDR method. Features with non-zero coefficients following regularization were retained for consideration.

As a complementary and confirmatory ranking approach, a random forest classifier was applied using the randomForest package (version 4.7-1.1) [29]. The forest was grown with 1000 trees (ntree = 1000) and the number of variables randomly sampled at each split (mtry) was tuned by LOOCV, evaluating mtry values of √*p*, *p*/3, and *p* (where *p* is the total number of input features), selecting the value minimizing out-of-bag [OOB] error. Class weights were set as being inversely proportional to class frequency to account for the imbalanced outcome distribution (classwt parameter). Given the small cohort size, random forest was used solely as a supportive feature-ranking method, rather than as a standalone predictive model.

Only features that were consistently ranked among the top variables by both methods, specifically, those with non-zero L2 coefficients and falling within the top quartile of random forest Gini importance, were retained for further biological consideration. The top-ranked associated features were visualized in a ranked dot plot using ggplot2 (version 3.4.4) [30], with the circle size representing FDR-adjusted *p*-values and color indicating the direction of association (higher in CR vs. higher in non-CR).

## Data Availability

The original contributions presented in this study are included in the article/Supplementary Material. Further inquiries can be directed to the corresponding authors.

## Supplementary Materials

The following supporting information can be downloaded at: https://www.mdpi.com/article/10.3390/ph19050671/s1. Figure S1. Plasma level of IL-2, IL-4, IL-9 and IL-7 in patients following CD19 CAR-T cell infusion. The concentrations of soluble human interleukins in the plasma of cancer patients (N = 18) were measured at baseline (day 0) and on days 7, 14, 21, 28, and 60 following CD19 CAR-T cell infusion. Quantification was performed simultaneously using flow cytometry with MACSPlex Capture Beads (Miltenyi Biotec Inc.). Figure S2. Hematological profiles of patients following CD19 CAR-T cell infusion. Whole blood samples were collected from 18 patients at baseline (day 0) and on days 7, 14, 21, and 28 after CD19 CAR-T cell infusion. Box plots display changes over time in red blood cells, hematocrit, hemoglobin, platelets, white blood cells, large unstained cells, neutrophils, lymphocytes, monocytes, eosinophils and basophils. Data are shown as median with interquartile range. Statistical significance compared to baseline is indicated (* *p* < 0.05, ** *p* < 0.01, and *** *p* < 0.001; Student’s *t*-test). Figure S3. Flow cytometry assessment of indicated cell lineages in PBMCs from patients with CD19 CAR-T cell infusion. Multiparameter flow cytometry analysis of indicated immune cell populations in peripheral blood from 18 patients at baseline (day 0) and on days 7, 14, 21, and 28 after CD19 CAR-T cell infusion. Peripheral blood mononuclear cells (PBMCs) from each individual sample were labeled after red cell lysis, using the reported panel of fluorophore-conjugated antibodies against lineage-specific cell surface markers in order to identify the B cells, NK cells, monocytes and macrophage cell subsets. Flow cytometry data are represented in distinct box plots for B-cells and plasma B-cells (A); natural killer (NK) cells (B); HLA-DR^high^ Classical and Intermediate Monocytes (C); M1 and M2 Macrophages (D). Table S1. Clinical and treatment characteristics of considered patients undergoing CD19 CAR-T cell therapy. DLBCL, diffuse large B-cell lymphoma; R/R, relapsed/refractory; NHL HG, high-grade non-Hodgkin lymphoma; CRS, cytokine release syndrome; ICANS, immune effector cell-associated neurotoxicity syndrome; ICE, immune effector cell encephalopathy; R-CHOP, R-COMP, R-DHAOX, R-DHAP = chemotherapy regimens; nd, not documented. Table S2. Targeted gene panel profiling in the considered patients undergoing CD19 CAR-T cell therapy. Columns correspond to individual patients with paired columns showing results for Day 0 and Day +28. Rows represent the specific genes analyzed. Mutations or variants detected at either timepoint are indicated; blank cells indicate no alteration identified. SNV, single nucleotide variant; MNV, multi-nucleotide variant; INDEL, small insertion and deletion. Table S3. Panel of cell surface markers and fluorophore-conjugated antibodies used for multiparameter flow cytometry analysis of T cell subsets in peripheral blood mononuclear cells (PBMCs) from cancer patients following CD19 CAR-T cell infusion. APC, allophycocyanin; SA, streptavidin; PE, phycoerythrin. Table S4. Panel of cell surface markers and fluorophore-conjugated antibodies used for multiparameter flow cytometry analysis of B cell and monocyte subsets in peripheral blood mononuclear cells (PBMCs) from cancer patients following CD19 CAR-T cell infusion. APC, allophycocyanin; PE, phycoerythrin. Table S5. Panel of cell surface markers and fluorophore-conjugated antibodies used for multiparameter flow cytometry analysis of macrophage and natural killer (NK) cell subsets in peripheral blood mononuclear cells (PBMCs) from cancer patients following CD19 CAR-T cell infusion. APC, allophycocyanin; PE, phycoerythrin. Table S6. Gating strategy for the identification of different cell subpopulations of peripheral blood mononuclear cells (PBMCs) from cancer patients, following CD19 CAR-T cell infusion. Fluorescence minus one (FMO) controls were applied to define gating boundaries for all markers. Doublets were excluded based on side scatter height (SSC-H) versus side scatter area (SSC-A) parameters, and dead cells were removed using a Live/Dead far-red DNA viability dye. Leukocytes were first identified as CD45^+^ events. Within this population, CAR-T cells were defined as CD19^−^CD3^+^ cells expressing the CAR construct (FMC63^+^). The remaining CD19^−^ CD3^+^ FMC63^−^ T-cell compartment was further subdivided into CD8^+^ T cells (CD4^−^CD8^+^), CD4^+^ T cells (CD4^+^CD8^−^), and regulatory T cells characterized as CD4^+^CD8^−^CD127^−^. Natural killer T (NKT) cells were identified as CD3^+^CD56^+^ cells. Natural killer (NK) cells were defined as CD3^−^CD56^+^CD19^−^ lymphocytes. B lymphocytes were gated as CD3^−^CD19^+^HLA-DR^+^ cells, and plasma cells were distinguished within the B-cell compartment by high CD38 expression (CD38^high^). Monocyte subsets were characterized according to CD14 and CD16 expression: classical monocytes (CD14^+^CD16^−^), intermediate monocytes (CD14^+^CD16^+^), and non-classical monocytes (CD14^dim^CD16^+^). The classical monocyte population was further stratified into HLA-DR^high^ and HLA-DR^low^ fractions. Within the CD14^+^CD16^+^CD163^−^ myeloid compartment, macrophage polarization states were evaluated by identifying CD80^+^ cells as M1-like macrophages and CD80^−^ cells as M2-like macrophages. Table S7. Exact log2 fold-change values and corresponding *p*-values for all evaluated features reported in Figure 2E.
