# Supplementary material for: Early Immune Signature Features, Including TLR2 and TLR4 Expression, Are Associated with Complete Remission After CD19 CAR-T Cell Therapy
Source: Pharmaceuticals (Basel). 2026 Apr 25;19(5):671. doi: 10.3390/ph19050671 (PMC13209260; doi:10.3390/ph19050671)
Supplement: Supplementary file 1 [file pharmaceuticals-19-00671-s001.zip › supplementary material.pdf]

Early Immune Signature Features, Including TLR2 and TLR4 Expression, Are Associated with Complete Remission After CD19 CAR-T Cell Therapy

Serena Di Iasio 1,†, Chiara Di Nunzio 1,†, Elisabetta De Santis 1, Concetta Stella 1, Daniela Valente 2, Dalila Salvatore 2, Emanuela Merla 2, Grazia Dell'Olio 2, Costanzo Padovano 1, Mattia Colucci 1, Gaja Bruno 1, Barbara Pasculli 3, Mario Caldarelli 4,5, Paola Parrella 3, Giovanni Gambassi 4,5, Rossella Cianci 4,5,*, Angelo M. Carella 2,* and Vincenzo Giambra 1

1 Hematopathology Unit, Institute for Stem Cell Biology, Regenerative Medicine and Innovative Therapeutics (ISBReMIT), Fondazione IRCCS "Casa Sollievo della Sofferenza", Viale Padre Pio, 7, 71013 San Giovanni Rotondo, Italy; s.diiasio@operapadrepio.it (S.D.I.); c.dinunzio@operapadrepio.it (C.D.N.); e.desantis@operapadrepio.it (E.D.S.); c.stella@operapadrepio.it (C.S.); costanzo.padovano@operapadrepio.it (C.P.); m.colucci@operapadrepio.it (M.C.); g.bruno@operapadrepio.it (G.B.); v.giambra@operapadrepio.it (V.G.)

2 Department of Hematology and Stem Cell Transplant Unit, Fondazione IRCCS "Casa Sollievo della Sofferenza", Viale Capuccini, 1, 71013 San Giovanni Rotondo, Italy; d.valente@operapadrepio.it (D.V.); d.salvatore@operapadrepio.it (D.S.); e.merla@operapadrepio.it (E.M.); g.dellolio@operapadrepio.it (G.D.)

3 Laboratory of Oncology, Fondazione IRCCS "Casa Sollievo della Sofferenza", Viale Cappuccini, 1, 71013 San Giovanni Rotondo, Italy; b.pasculli@operapadrepio.it (B.P.); pparrella@operapadrepio.it (P.P.)

4 Department of Translational Medicine and Surgery, Catholic University of Sacred Heart, Largo Agostino Gemelli, 8, 00168 Rome, Italy; mario.caldarelli01@icatt.it (M.C.); giovanni.gambassi@unicatt.it (G.G.)

5 Fondazione Policlinico Universitario A. Gemelli, Istituto di Ricerca e Cura a Carattere Scientifico (IRCCS), Largo Agostino Gemelli, 8, 00168 Rome, Italy

* Correspondence: rossella.cianci@unicatt.it (R.C.); am.carella@operapadrepio.it (A.M.C.)

† These authors contributed equally to this work.

Supplemental Information including:

Figures S1 to S3

Table Legends S1 to S2

Tables S3 to S7

Supplementary Figures

Fig. S1.

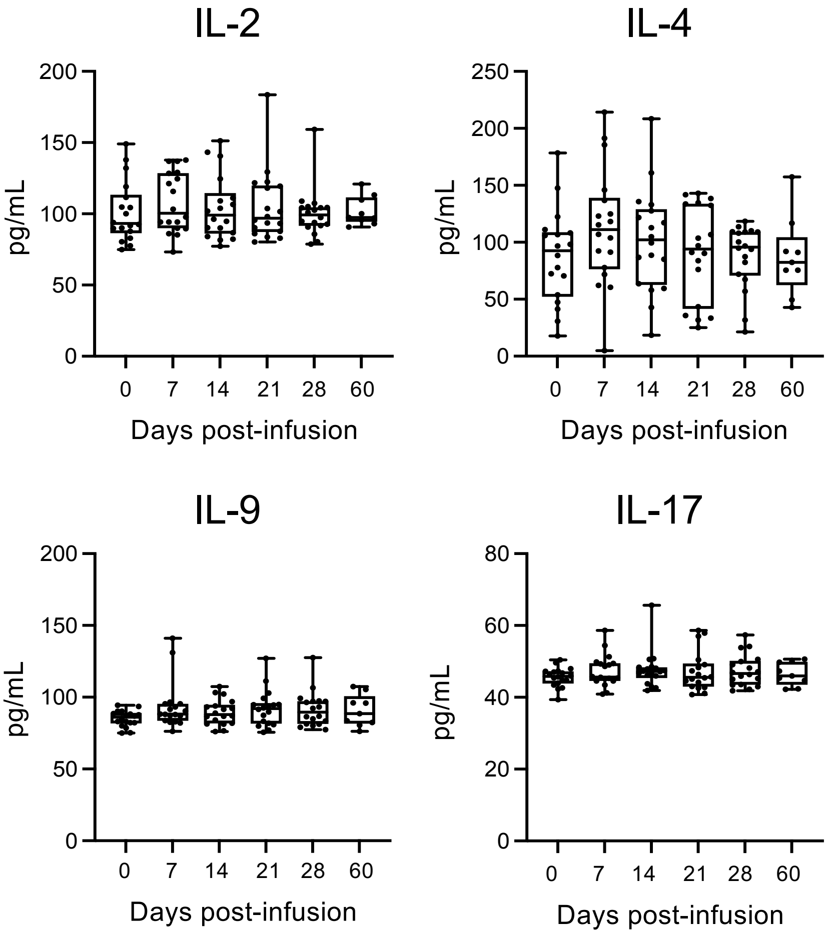

Figure S1. Plasma level of IL-2, IL-4, IL-9 and IL-7 in patients following CD19 CAR-T cell infusion.

The concentrations of soluble human interleukins in the plasma of cancer patients (N = 18) were measured at baseline (day 0) and on days 7, 14, 21, 28, and 60 following CD19 CAR-T cell infusion. Quantification was performed simultaneously using flow cytometry with MACSPlex Capture Beads (Miltenyi Biotec Inc.).

Fig. S2.

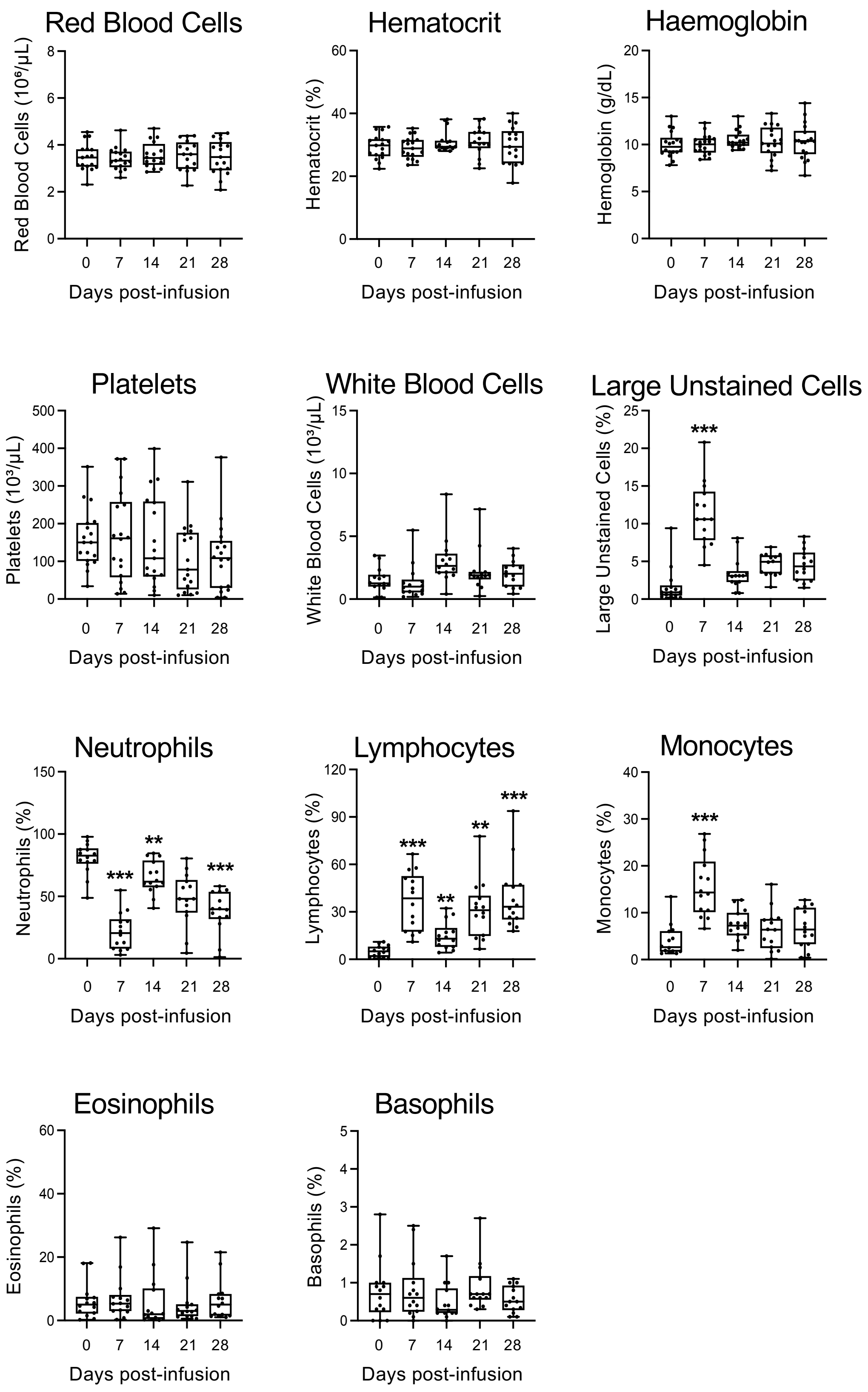
Red Blood Cells
Red Blood Cells (10⁶/µL)
Hematocrit
Hematocrit (%)
Haemoglobin
Hemoglobin (g/dL)
Platelets
Platelets (10³/µL)
White Blood Cells
White Blood Cells (10³/µL)
Large Unstained Cells
Large Unstained Cells (%)
Neutrophils
Neutrophils (%)
Lymphocytes
Lymphocytes (%)
Monocytes
Monocytes (%)
Eosinophils
Eosinophils (%)
Basophils
Basophils (%)
Days post-infusion
0 7 14 21 28
***
**

Figure S2. Hematological profiles of patients following CD19 CAR-T cell infusion. Whole blood samples were collected from 18 patients at baseline (day 0) and on days 7, 14, 21, and 28 after CD19 CAR-T cell infusion. Box plots display changes over time in red blood cells, hematocrit, hemoglobin, platelets, white blood cells, large unstained cells, neutrophils, lymphocytes, monocytes, eosinophils and basophils. Data are shown as median with interquartile range. Statistical significance compared to baseline is indicated (*$^*p < 0.05$, $^{**}p < 0.01$, $^{***}p < 0.001$; Student's t-test*).

Fig. S3.

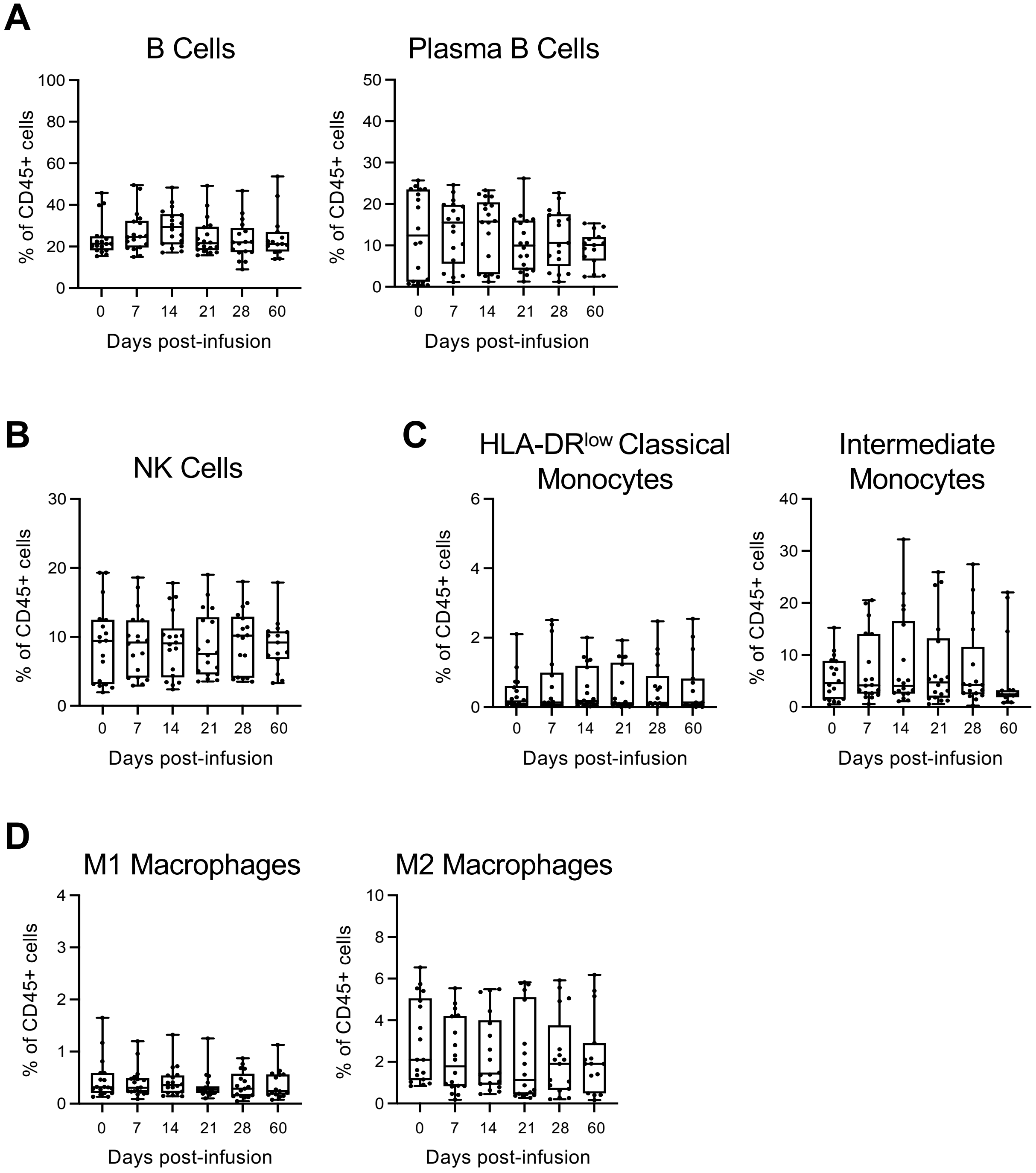
A
B Cells
Plasma B Cells
% of CD45+ cells
Days post-infusion
0
7
14
21
28
60
B
NK Cells
C
HLA-DR^low Classical Monocytes
Intermediate Monocytes
D
M1 Macrophages
M2 Macrophages

Figure S3. Flow cytometry assessment of indicated cell lineages in PBMCs from patients with CD19 CAR-T cell infusion.

Multiparameter flow cytometry analysis of indicated immune cell populations in peripheral blood from 18 patients at baseline (day 0) and on days 7, 14, 21, and 28 after CD19 CAR-T cell infusion. Peripheral blood mononuclear cells (PBMCs) from each individual sample were labelled after red cell lysis using the reported panel of fluorophore-conjugated antibodies against lineage-specific cell surface markers in order to identify the B cells, NK cells, monocytes and macrophage cell subsets. Flow cytometry data are represented in distinct box plots for B-cells and plasma B-cells (**A**); Natural Killer (NK) cells (**B**); HLA-DR^{high} Classical and Intermediate Monocytes (**C**); M1 and M2 Macrophages (**D**).

Supplementary Table Legends

Table S1. Clinical and treatment characteristics of considered patients undergoing CD19 CAR-T cell therapy. DLBCL, diffuse large B-cell lymphoma; R/R, relapsed/refractory; NHL HG, high-grade non-Hodgkin lymphoma; CRS, cytokine release syndrome; ICANS, immune effector cell–associated neurotoxicity syndrome; ICE, immune effector cell encephalopathy; R-CHOP, R-COMP, R-DHAOX, R-DHAP = chemotherapy regimens; nd, not documented.

Table S2. Targeted gene panel profiling in the considered patients undergoing CD19 CAR-T cell therapy. Columns correspond to individual patients with paired columns showing results for Day 0 and Day +28. Rows represent the specific genes analyzed. Mutations or variants detected at either timepoint are indicated; blank cells indicate no alteration identified. SNV, single nucleotide variant; MNV, multi-nucleotide variant; INDEL, small insertion and deletion.

Supplementary Tables

Table S3.

Flow Cytometry Panel for T Cell Subsets

Laser	Detector	Fluorophore	Marker	Antibody Clone	Company	Catalog #
405	448/59	Super Bright 436	CD3	SK7	eBioscience™	62-0036-41
	525/50	eFluor 506	CD45	HI30	eBioscience™	69-0459-41
	710/45	Super Bright 702	CD4	SK3	eBioscience™	67-0047-42
	755 LP	Super Bright 780	CD8	53-6.7	eBioscience™	78-0081-82
488	513/26	Alexa Fluor 488	CD282 (TLR2)	TL2.1	eBioscience™	53-9922-42
	795/70	Biotin +SA-PE-Cyanine7	CAR FMC63	REA1297	MiltenyiBiotec	130-127-345
561	579/16	PE	CD127	eBioRDR5	eBioscience™	12-1278-42
640	671/30	APC	CD284 (TLR4)	HTA125	eBioscience™	17-9917-42
	722/44	Alexa Fluor 700	CD19	SJ25C1	eBioscience™	MHCD1929
	795/70	APC-eFluor 780	Live / Dead		eBioscience™	L34975

Table S3. Panel of cell surface markers and fluorophore-conjugated antibodies used for multiparameter flow cytometry analysis of T cell subsets in peripheral blood mononuclear cells (PBMCs) from cancer patients following CD19 CAR-T cell infusion. APC, allophycocyanin; SA, streptavidin; PE, phycoerythrin.

Table S4.

Flow Cytometry Panel for B Cell and Monocyte Subsets

Laser	Detector	Fluorophore	Marker	Antibody Clone	Company	Catalog #
405	448/59	Super Bright 436	CD3	SK7	eBioscience™	62-0036-41
	525/50	eFluor 506	CD45	HI30	eBioscience™	69-0459-41
	620/29	Qdot 605	HLA-DR	Tü36	eBioscience™	Q10052
	710/45	Super Bright 702	CD4	SK3	eBioscience™	67-0047-42
	755 LP	Super Bright 780	CD8	53-6.7	eBioscience™	78-0081-82
488	513/26	Alexa Fluor 488	CD282 (TLR2)	TL2.1	eBioscience™	53-9922-42
	795/70	PE-Cyanine7	CD16	eBioCB16	eBioscience™	25-0168-42
561	579/16	PE	CD127	eBioRDR5	eBioscience™	12-1278-42
	614/20	PE-eFluor 610	CD14	61D3	eBioscience™	61-0149-42
	692/75	PE-Cyanine5	CD38	HIT2	eBioscience™	15-0389-42
640	671/30	APC	CD284 (TLR4)	HTA125	eBioscience™	17-9917-42
	722/44	Alexa Fluor 700	CD19	SJ25C1	eBioscience™	MHCD1929
	795/70	APC-eFluor 780	Live / Dead		eBioscience™	L34975

Table S4. Panel of cell surface markers and fluorophore-conjugated antibodies used for multiparameter flow cytometry analysis of B cell and monocyte subsets in peripheral blood mononuclear cells (PBMCs) from cancer patients following CD19 CAR-T cell infusion. APC, allophycocyanin; PE, phycoerythrin.

Table S5.

Flow Cytometry Panel for Macrophage and NK Cell Subsets

Laser	Detector	Fluorophore	Marker	Antibody Clone	Company	Catalog #
405	448/59	Super Bright 436	CD3	SK7	eBioscience™	62-0036-41
	525/50	eFluor 506	CD45	HI30	eBioscience™	69-0459-41
	620/29	Super Bright 600	CD163	eBioGHI/61	eBioscience™	63-1639-42
	710/45	Super Bright 702	CD80	2D10.4	eBioscience™	67-0809-42
488	513/26	Alexa Fluor 488	CD282 (TLR2)	TL2.1	eBioscience™	53-9922-42
	795/70	PE-Cyanine7	CD16	eBioCB16	eBioscience™	25-0168-42
561	579/16	PE	RANK	9A725	eBioscience™	MA1-41015
	614/20	PE-eFluor 610	CD14	61D3	eBioscience™	61-0149-42
	692/75	PE-Cyanine5	CD56	CMSSB	eBioscience™	15-0389-42
640	671/30	APC	CD284 (TLR4)	HTA125	eBioscience™	17-9917-42
	722/44	Alexa Fluor 700	CD19	SJ25C1	eBioscience™	MHCD1929
	795/70	APC-eFluor 780	Live / Dead		eBioscience™	L34975

Table S5. Panel of cell surface markers and fluorophore-conjugated antibodies used for multiparameter flow cytometry analysis of macrophage and natural killer (NK) cell subsets in peripheral blood mononuclear cells (PBMCs) from cancer patients following CD19 CAR-T cell infusion. APC, allophycocyanin; PE, phycoerythrin.

Table S6.

Cell Type	Gating Strategy
CAR-T cells	CD45+ CD19- CD3+ CAR/FMC63+
B cells	CD45+ CD3- CD19+ HLA-DR+
Plasma B cells	CD45+ CD3- CD19+ HLA-DR+CD38^{high}
NK cells	CD45+ CD3- CD56+ CD19-
NK T-like cells	CD45+ CD3+ CD56+
CD4 + T cells	CD45+ CD19- CAR/FMC63- CD3+ CD4+ CD8-
CD4 Regulatory T cells	CD45+ CD19- CAR/FMC63- CD3+ CD4+ CD8- CD127-
CD8 + T cells	CD45+ CD19- CAR/FMC63- CD3+ CD8+ CD4-
Classical Monocytes	CD45+ CD14+ CD16-
HLA-DR^{high} Classical Monocytes	CD45+ CD14+ CD16- HLA-DR^{high}
HLA-DR^{low} Classical Monocytes	CD45+ CD14+ CD16- HLA-DR^{low}
Intermediate Monocytes	CD45+ CD14+ CD16+
Non-Classical Monocytes	CD45+ CD14^{dim} CD16-
M1 Macrophages	CD45+ CD14+ CD16+ CD80+ CD163-
M2 Macrophages	CD45+ CD14+ CD16+ CD80- CD163-

Table S6. Gating strategy for the identification of different cell subpopulations of peripheral blood mononuclear cells (PBMCs) from cancer patients following CD19 CAR-T cell infusion. Fluorescence Minus One (FMO) controls were applied to define gating boundaries for all markers. Doublets were excluded based on side scatter height (SSC-H) versus side scatter area (SSC-A) parameters, and dead cells were removed using a Live/Dead far-red DNA viability dye. Leukocytes were first identified as CD45^{+} events. Within this population, CAR-T cells were defined as CD19^{-} CD3^{+} cells expressing the CAR construct (FMC63^{+}). The remaining CD19^{-} CD3^{+} FMC63^{-} T-cell compartment was further subdivided into CD8^{+} T cells (CD4^{-}CD8^{+}), CD4^{+} T cells (CD4^{+}CD8^{-}), and regulatory T cells characterized as CD4^{+}CD8^{-}CD127^{-}. Natural killer T (NKT) cells were identified as CD3^{+}CD56^{+} cells. Natural killer (NK) cells were defined as CD3^{-}CD56^{+}CD19^{-} lymphocytes. B lymphocytes were gated as CD3^{-}CD19^{+}HLA-DR^{+} cells, and plasma cells were distinguished within the B-cell compartment by high CD38 expression (CD38^{high}). Monocyte subsets were characterized according to CD14 and CD16 expression: classical monocytes (CD14^{+}CD16^{-}), intermediate monocytes (CD14^{+}CD16^{+}), and non-classical monocytes (CD14^{dim}CD16^{+}). The classical monocyte population was further stratified into HLA-DR^{high} and HLA-DR^{low} fractions. Within the CD14^{+}CD16^{+}CD163^{-} myeloid compartment, macrophage polarization states were evaluated by identifying CD80^{+} cells as M1-like macrophages and CD80^{-} cells as M2-like macrophages.

Table S7.

Rank	Feature	Timepoint	Log2FC	Adjusted p-value
1	Intermediate Monocytes (%)	d7	4.0	0.0123
2	Intermediate Monocytes (%)	d0	2.9	0.0054
3	TLR2 (MFI) in M1 macrophages	d7	2.2	0.0203
4	TLR2 (MFI) in M1 macrophages	d0	2.1	0.0193
5	Classical Monocytes (%)	d0	2.1	0.0394
6	TLR2 (MFI) in M2 macrophages	d7	1.8	0.0498
7	IL-2 (pg/mL)	d0	1.1	0.0503
8	IFN-γ (pg/mL)	d7	0.75	0.0193
9	TLR4 (MFI) in Non-Classical Monocytes	d7	0.65	0.0434
10	TLR4 (MFI) in Intermediate Monocytes	d7	0.70	0.0103
11	TLR2 (MFI) in Non-Classical Monocytes	d7	0.60	0.0393
12	TLR4 (MFI) in CD4+ Tregs	d7	0.65	0.0208
13	TLR2 (MFI) in CD4+ Tregs	d7	0.65	0.0119
14	TLR2 (MFI) in CD4+ T cells	d7	0.68	0.0047
15	TLR2 (MFI) in Intermediate Monocytes	d0	0.45	0.0024
16	TLR2 (MFI) in Intermediate Monocytes	d7	0.42	0.0003

Table S7. Exact log2 fold-change values and corresponding p-values for all evaluated features reported in Figure 2E.
